# Biodegradable magnesium barrier membrane used for guided bone regeneration in dental surgery

**DOI:** 10.1016/j.bioactmat.2021.11.018

**Published:** 2021-11-29

**Authors:** Patrick Rider, Željka Perić Kačarević, Akiva Elad, Drazen Tadic, Daniel Rothamel, Gerrit Sauer, Fabien Bornert, Peter Windisch, Dávid Botond Hangyási, Balint Molnar, Emely Bortel, Bernhard Hesse, Frank Witte

**Affiliations:** a Department of Prosthodontics, Geriatric Dentistry and Craniomandibular Disorders, Charité – Universitätsmedizin Berlin, Aßmannshauser Straße 4–6, 14197, Berlin, Germany; b Botiss Biomaterials AG, Ullsteinstrasse 108, 12109, Berlin, Germany; c Department of Anatomy Histology, Embryology, Pathology Anatomy and Pathology Histology, Faculty of Dental Medicine and Health, University of Osijek, Osijek, 31000, Croatia; d CMF Surgery, Johannes BLA Hospital, Mönchengladbach, Germany; e Dental Clinic, University of Strasbourg, France; f Department of Periodontology, Semmelweis University, Budapest, Hungary; g Xploraytion GmbH, Bismarkstrasse 11, Berlin, Germany; h Biotrics Bioimplants AG, Ullsteinstrasse 108, 12109, Berlin, Germany

**Keywords:** GBR, Guided Bone Regeneration, Magnesium, Biodegradable, Implant, GBR, Bone healing, Soft tissue healing

## Abstract

Barrier membranes are commonly used as part of the dental surgical technique guided bone regeneration (GBR) and are often made of resorbable collagen or non-resorbable materials such as PTFE. While collagen membranes do not provide sufficient mechanical protection of the covered bone defect, titanium reinforced membranes and non-resorbable membranes need to be removed in a second surgery. Thus, biodegradable GBR membranes made of pure magnesium might be an alternative. In this study a biodegradable pure magnesium (99.95%) membrane has been proven to have all of the necessary requirements for an optimal regenerative outcome from both a mechanical and biological perspective. After implantation, the magnesium membrane separates the regenerating bone from the overlying, faster proliferating soft tissue. During the initial healing period, the membrane maintained a barrier function and space provision, whilst retaining the positioning of the bone graft material within the defect space. As the magnesium metal corroded, it formed a salty corrosion layer and local gas cavities, both of which extended the functional lifespan of the membrane barrier capabilities. During the resorption of the magnesium metal and magnesium salts, it was observed that the membrane became surrounded and then replaced by new bone. After the membrane had completely resorbed, only healthy tissue remained. The *in vivo* performance study demonstrated that the magnesium membrane has a comparable healing response and tissue regeneration to that of a resorbable collagen membrane. Overall, the magnesium membrane demonstrated all of the ideal qualities for a barrier membrane used in GBR treatment.

## Highlights

• First report on a biodegradable metallic barrier membrane for use in oral surgery is presented.
• The mechanical stability of the metallic barrier membrane provides a careful shielding of the augmented bone defect.
• Full resorption of metallic barrier membrane and bone healing is completed long before current standards for second surgical patient treatment.

## Graphical abstract

**Figure:**
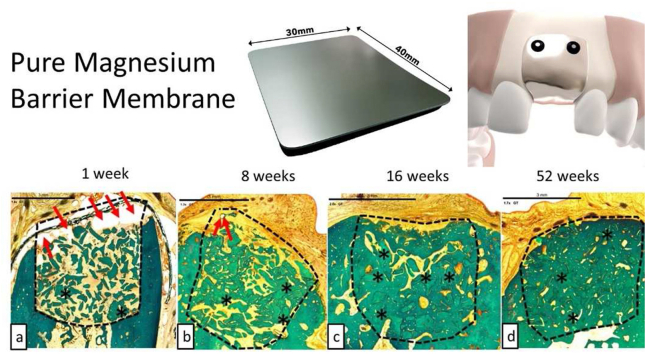

## 1 Introduction

Barrier membranes are commonly used as part of the dental surgical technique guided bone regeneration (GBR). The barrier membrane is positioned to seclude gingival soft tissues from migrating into the space created by bony defects, thus enabling new bone to populate the area and restore functionality [1,2]. Both resorbable and non-resorbable membrane options exist, yet regardless of the material, certain criteria must be fulfilled including biocompatibility, non-immunogenicity, non-toxicity, space making ability, cell-occlusion, tissue integration and clinical manageability [3,4]. As all membranes show advantageous and disadvantageous properties, the choice of membrane should be based on the necessary biological properties and treatment requirements [4].

In the early 1980s, barrier membranes were introduced to the technique of GBR for the seclusion of bony defects [5]. These membranes were non-resorbable and primarily made from polytetrafluoroethylene (PTFE). Non-resorbable membranes provide the advantage of maintaining their shape and structure for the duration of the treatment, yet require a second surgical procedure for their removal. In response to this disadvantage, resorbable membranes were developed [3]. From a clinical perspective, degradable membranes are preferable as a second surgery is unnecessary, reducing the risk of morbidity and trauma for the patient, as well as overall procedural costs.

Resorbable membranes are available as synthetic and organic variants, and are generally composed of collagen [6] or the synthetic polymers: poly lactic acid (PLA), poly glycolic acid (PGA) and their copolymers [7,8]. Although membranes manufactured from PLA and PGA are successfully applied in GBR procedures, their biodegradation may lead to a local increase in acidity that can create an unfavorable environment for tissue regeneration [[9], [10], [11]]. Depending on the polymer, synthetic bioresorbable membranes may induce a mild to severe inflammatory reaction that attracts lymphocytes and multinucleated giant cells [[12], [13], [14]]. The inflammatory reaction combined with a reduction in pH can provoke the degradation of newly formed bone [13,15]. Moreover, synthetic polymer membranes are generally not as biologically active as natural polymers [16], and their lack of rigidity and stability have also been reported as a disadvantage [17].

Collagen is the most frequently used resorbable barrier membrane [18] and has been investigated for use in GBR since the late 1980s [19]. Although collagen membranes present an improved bioactivity in comparison to the synthetic alternatives, there is the potential issue for the membrane to collapse into the defect, thereby restricting the volume available for the regenerating bone [20,21].

It has previously been reported that resorbable membranes with a high stiffness are able to offer similar levels of bone formation as non-resorbable membranes [4,22]. It has also been shown that soft tissue responses are improved when using a resorbable membrane with better tissue integration when compared to non-resorbable membranes [[22], [23], [24]]. Therefore, applying a stiff resorbable membrane for GBR treatment could prove beneficial for the regenerative outcome.

To address the aforementioned issues with the current GBR membranes, an alternative material has been investigated. Magnesium is a biodegradable metal that is resorbed by the human body without toxic residuals [[25], [26], [27]] and has yet to be used in regenerative dentistry. Magnesium ions (Mg^2+^) are a naturally occurring component in the human organism and are responsible for many physiological processes [[28], [29], [30]]. Due to the natural prevalence of Mg^2+^ in the body, there is already an effective method for their excretion via the kidneys and intestine [[29], [30], [31]].

Magnesium and its alloys have shown excellent biocompatibility and are currently used for cardiovascular stents [[32], [33], [34]], tracheal stents [35], orthopedic screws [25,36,37], osteosynthesis systems (cranio-maxillofacial surgery) [38,39] and bone repair materials, such as fracture plates [40]. Magnesium implants provide a mechanical stability that is more similar to bone than other metallic implants [41]. Yet once implanted, magnesium implants begin to corrode and are completely resorbed [36,[42], [43], [44]]. As the corrosion process occurs within the body, it is synonymously referred to as biodegradation, hence magnesium is a biodegradable metal.

During the corrosion process, the metallic magnesium is oxidized and magnesium ions are released as corrosion products (Eq. (1)). At the same time, hydrogen atoms in water molecules are reduced (Eq. (2)), releasing molecular hydrogen as an additional corrosion product. A hydroxide layer is formed from the interaction of the magnesium metal and water molecules (Eq. (3)), which is deposited on the surface of the magnesium implant [29]. These layers are susceptible to corrosion, especially in the presence of anions (Eq. (4)). The magnesium hydroxide present in the corrosion layer is attacked by soluble chloride ions when sodium chloride concentrations are higher than 30 mM, which is easily reached in physiological solutions or in the human body [45]. As a result, the protective Mg(OH)_2_ layer is dissolved, at least locally, enabling the corrosion process to continue. This principle leads to the complete corrosion of the magnesium implant [46].

(Eq.1) Oxidation: Mg → Mg^2+^ + 2e^−^

(Eq.2) Reduction: 2H_2_O + 2e^−^ → 2OH^−^ + H_2_ ↑

(Eq.3) Hydroxide Formation: Mg + 2H_2_O → Mg(OH)_2_ + H_2_ ↑

(Eq.4) Breakdown of the Oxide Layer: Mg(OH)_2_ + 2Cl^-^ → MgCl_2_ + 2OH^−^

During the corrosion process, free hydroxyl ions are released (Eq. (2)). These ions shift the pH within the immediate vicinity of the implant into the alkaline range. Therefore, the occurrence of inflammation and osteolysis as a result of an acidic pH shift, as can be observed with synthetic polymers [13,15], is highly unlikely. Negative influence on bone regeneration due to the alkaline pH is also unlikely, as bone substitute materials composed of pure hydroxyapatite degrade with a slight alkaline pH and have been applied successfully in bone regeneration procedures for many years [47].

As part of the corrosion process, hydrogen is produced in a one to one stoichiometric relation (Eq. (3)), which often causes the formation of gas cavities around the implant. However, the gas cavities are typically only present during the corrosion of the metallic phase of the magnesium implant and are observed to resolve spontaneously without any treatment and without negatively influencing bone formation [30,36,[42], [43], [44],^48^,^49^].

A magnesium membrane has been developed and tested as reported in this article. The membrane is intended to provide a barrier function similar to other resorbable membranes in GBR, yet with greater mechanical stability to enhance the protection of the defect void.

## 2 Materials and methods

### 2.1 Tested material

**Fig. 1:**
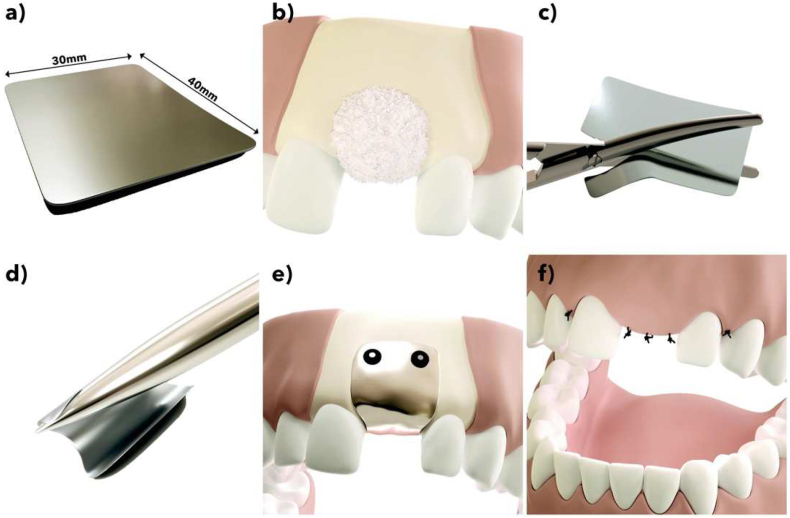
The magnesium barrier membrane (NOVAMag® membrane, botiss biomaterials GmbH, Germany) and its insertion protocol. a) The magnesium membrane. b) Before placement of the membrane, the defect sites is prepared for augmentation and filled with an appropriate bone augmentation material. c) The membrane is cut to shape using a pair of scissors (NOVAMag® scissors). d) The membrane is then bent to fit the defect site contours. e) The membrane is fixated from both the buccal and palatial/lingual sides. f) The mucoperiosteal flap is sutured closed over the membrane for closed wound healing.

The magnesium membranes (NOVAMag® membrane, botiss biomaterials GmbH, Germany) (Fig. 1) used in this study were produced at biotrics bioimplants AG (Berlin, Germany) from pure magnesium (99.95%). The membranes were made from a hot-rolled magnesium sheet with a 250 μm thickness. Initially, the magnesium sheet underwent a grinding process to remove surface impurities, after which, it was cut to shape using a stamping press and die. Each membrane was then etched using a company-specific process. After the etching process, the membranes were sterilized using gamma irradiation with a total dosage of 30 kGy. The membranes are 30 × 40 mm, with a thickness of 140 μm, and an average weight of 280 mg. The corners of the membrane are rounded with a 4 mm radius. Due to its smooth surface, it has a mirror-like shine.

### 2.2 Magnesium purity

The chemical composition of the magnesium membrane was analyzed from 3 separate batches of its raw material. To determine the content of specified elements (Al, As, Be, Ca, Cd, Cr, Cu, Fe, Hg, Mn, Na, Ni, Pb, Si, V, Zn), batches were analyzed using atomic emission spectroscopy with inductive-coupled plasma (ICP-OES), mass spectrometry with inductive-coupled plasma (ICP-MS) and atomic absorption spectroscopy (AAS), which were performed at ChemiLytics GmbH. The respective detection limits for each method are: 10 ppm for the ICP-MS and the AAS; and 30 ppm for the ICP-OES.

### 2.3 Material microstructure

Metallurgical samples of the pure magnesium membrane were embedded in resin and were ground by silicon carbide emery paper with wet grinding from 800 to 2500 before polishing with water-free colloidal silica solution (0.2 μm particle size). The etching agent used was picric acid based, containing 10 ml of acetic acid, 4.2 g of picric acid, 20 ml H_2_O and 50 ml ethanol. The images were acquired by optical microscopy (Leica DMI 5000, Nomarski contrast, polarized light) and grain size analysis was performed according to linear intercept method according to ASTM:E112-13 (2013).

### 2.4 Initial mechanical properties of tested material

#### 2.4.1 Tensile testing

**Fig. 2:**
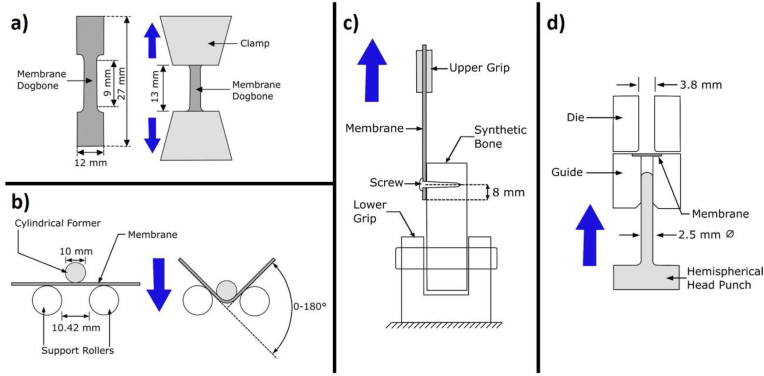
Test setups for the mechanical evaluation of the magnesium membrane (colored dark grey in schematics). In the schematics, the light grey colored pieces of equipment move in the direction of the blue arrow, whilst the white colored pieces remain in a fixed position. a) Dogbone shape and tensile test setup for the magnesium membrane. b) Membrane bending test setup, based on the ISO 7438-05 (Metallic materials – Bend test). c) Setup testing for the resistance of the membrane to tensile loads at its anchoring point. This setup is based on the requirements of ASTM F564. d) Test setup for the “Small punch test”, based on the requirements of ASTM F2183-02. (For interpretation of the references to color in this figure legend, the reader is referred to the Web version of this article.)

Tensile testing was used to measure the tensile stress at yield, percentage elongation at yield, the maximum tensile stress, percentage elongation at maximum stress, percentage elongation at fracture, and the modulus of elasticity. Magnesium membranes were cut into dog-bone shaped test samples (Fig. 2a) using a stamping press and had their width and thickness measured at three different locations within the test region to an accuracy of 0.005 mm. The dog-bone samples were loaded into a Universal testing machine (5569A Series, Instron, Norwood, MA-USA) equipped with a 50 kN load cell (2525-802 Series, Instron, Norwood, MA-USA) with a distance between the clamps of 13 mm. In accordance with the ISO 6892-1, a tensile load was applied at a rate of 0.00025 s^−1^ multiplied by the gauge length, which in this instance was 9 mm. Elongation was measured using a video-extensometer (Advanced Video Extensometer, Instron, Norwood, MA-USA) with an accuracy of ±2.5 μm. The test was repeated 10 times under room temperature conditions (23.0 °C). The test protocol aligns with the requirements of ISO 6892-1:2017-02.

#### 2.4.2 Resistance to tensile loads at anchoring point

The magnesium membrane was tested for its ability to withstand tensile loads applied to its anchoring point. A collagen membrane (Bio-Gide®, Geistlich AG) was also tested in a dry state to provide reference values. Membranes were cut into 10 mm strips and secured to a synthetic bone block (grade 40, Sawbones Europe AB, Sweden) (the specifications of the synthetic bone block met the requirements of ASTM F1839), with a fixation screw (Pro-fix Precision Fixation System, Osteogenics) at a singular anchoring point. The distance between the anchor point and the end of the membrane was 8 mm. A schematic for the test setup is shown in Fig. 2c. Tensile loads were applied at a rate of 1 mm s^−1^ using a Universal testing machine (5569A Series, Instron, Norwood, MA-USA) until failure occurred and was repeated six times per group. This test protocol is based on the specifications of ASTM F564-17.

#### 2.4.3 Bending test

The ability to shape the membrane was evaluated using a static bending test and was repeated for 6 membranes. The bending test was performed using a Universal testing machine (5544 Series, Instron, Norwood, MA-USA). Each sample was placed onto two support rollers, each with a diameter of 10 mm and a separation of 10.42 mm, according to the test setup schematic shown in Fig. 2b. A static compressive load was applied to the top of the samples between the support rollers by a cylindrical former that had a dimeter of 10 mm and a loading rate of 1 mm s^−1^. Loading was stopped once a bending angle of 180° was reached. After the test, a visual inspection of the samples for characteristic signs of damage was performed using a digital microscope (VHX S550E Series, Keyence) with a 10x magnification. The static bending test was performed according to the requirements of ISO 7438:2005, and the visual inspection of the membrane surface was performed according to ASTM WK57407.

### 2.5 *In-vitro* corrosion tests

#### 2.5.1 Corrosion test set-up

Magnesium membranes were submerged in a physiological solution of Hank's Balanced Salt Solution (HBSS) with the addition of 0.35 g L^−1^ NaHCO3. 0.2 mL of solution was used for every 1 mm^2^ surface area of the membrane, which is in accordance with ASTM G31. Each magnesium membrane was stored in individual borosilicate glass containers filled with 282 mL of test fluid. The membranes were incubated at 37 ± 2 °C with a 90% relative humidity and a CO_2_ range between 1 and 1.5%. The pH of the solution was set at 7.4 and frequently monitored (pH/redox/temperature measuring device with data logger, Greisinger electronic GmbH, Type GMH 3551) and regulated using the environmental CO_2_ level. Five membranes were used for each of the corrosion time points, which were: 0 h, 24 h, 48 h, 72 h, and 168 h. Before subsequent analysis of the membranes was performed, the samples were rinsed and immersed in acetone to remove excess electrolyte solution.

#### 2.5.2 Mass loss

Prior to testing, each membrane was weighed (High precision balance, Sartorius, Type BP 211-D) to an accuracy or 0.01 mg. Each membrane was then corroded for a set period of time using the corrosion test set up (see section 2.5.1). After the membranes had been rinsed, they were subsequently cleaned with chromic acid to remove the corrosion products. The membranes were weighed again and mass loss and corrosion rates calculated.

Corrosion rates (mm y^−1^) for each time point were calculated using the following equation:

(Eq. 5) CorrosionRate=(K×W)/(A×T×D)

where $K=8.76\times 10^{4}$, W is mass loss in grams, A is the area of the membrane in cm^2^ (24 cm^2^), T is the time of exposure in hours, and D is the material density (1.74 g cm^−3^).

#### 2.5.3 Small punch test

A small punch test was performed to determine the changes in mechanical behavior of the magnesium membrane as it corroded. Before performing the *in vitro* corrosion test, five magnesium membrane samples and five collagen membrane samples (Bio-Gide, Geistlich) were tested. At each time point of the *in vitro* corrosion test, five magnesium membrane samples were tested within 1 h of their retrieval. Prior to performing the small punch test, the membranes were cut using a hollow punch into 8 mm diameter disks. In a dry state, the disks were individually loaded into a testing device (setup based on ASTM F2183-02, see Fig. 2d) consisting of a hemispherical head punch (2.5 mm diameter), a die, and a guide for the punch. Each sample was clamped between two metal fixtures and a static compressive load of 0.5 mm min^−1^ was applied perpendicular to the membrane surface using the punch and a Universal testing machine (5544 Series, Instron, Norwood, MA-USA) equipped with a force transducer (2580-107 Series, Instron, Norwood, MA-USA) and a displacement transducer (5544 Series, Instron, Norwood, MA-USA).

### 2.6 Biological safety assessment

Biological safety was analyzed through multiple cell and animal tests. All animal biocompatibility tests performed for the biological safety evaluation were approved of by the NAMSA Ethical Committee and by the Ministry of Education, Higher Education and Research. Each procedure is part of a project authorization (Authorization numbers 05306.03A and APAFIS#14881-2018021415456720 v2) that is reviewed every five years. Any significant changes to the procedures were approved prior to conduct. NAMSA is an AAALAC international accredited facility and is registered with the French Department of Agriculture for animal housing, care and investigations. The tests were performed according to the current ISO 10993 series, unless stated elsewhere.

#### 2.6.1 Cell media extracts

Eagle Minimum Essential Medium (EMEM) (reference M2279, Sigma-Aldrich), supplemented with 10% (v/v) Fetal bovine serum (FBS) (reference F7524, Sigma-Aldrich), l-glutamine (reference G7513, Sigma-Aldrich) (>2 mM) and antibiotics (2% (v/v) Penicillin (100 units/mL), Streptomycin (>100 μg/mL) (reference P4458, Sigma-Aldrich), and 1% (v/v) Amphotericin B(2.5–3 μg/mL) (reference A2942, Sigma-Aldrich)) was used as an extraction medium. Using an extraction ratio of 6 cm^2^ membrane surface area to 1 mL of extraction medium, the magnesium membrane was submerged in the extraction medium and maintained at a temperature of 37 °C for 72 h. During the extraction process, the extract was continuously agitated (50 revolutions per minute on a tube roller). Following extraction, the extract was used immediately for testing. The extract was diluted to concentrations of 50%, 25% and 12.5% (v/v) using EMEM.

In accordance with ISO 10993-5, a negative, blank, and positive control were prepared as follows. A negative control of a high density polyethylene (HDPE) sheet (Hatano Research Institute, Food and Drug Safety Center Grade) was prepared using an extraction ratio of 6 cm^2^ sample surface area to 1 mL of extraction medium. The negative control was not diluted.

A control blank was prepared the same way as the test extract, however without using the magnesium membrane. The purpose of the blank control is to assess possible falsifying effects of the extraction vessel, the extraction medium and the extraction process.

A positive control of a polyurethane film containing 0.1% zinc diethyldithiocarbamate (Hatano Research Institute, Food and Drug Safety Center) was prepared using an extraction ratio of 6 cm^2^ sample surface area to 1 mL of extraction medium. The positive control was diluted with EMEM to concentrations of 25%, 20%, 15%, 10% and 3% (v/v).

#### 2.6.2 Polar and apolar extracts

A 0.9% Sodium chloride (SC) (NaCl, CAS No. 7647-14-5) was used as a polar extraction vehicle and sesame oil (SO) (CAS No. 8008-74-0) was used as an apolar extraction vehicle. Water (WFI) (CAS No. 7732-18-5) was additionally used as an extraction media. Using an extraction ratio of 6 cm^2^ membrane surface area to 1 mL of extraction media, the magnesium membrane was submerged in the extraction media and maintained at a temperature of 50 °C for 72 h. During the extraction process, the extract was continuously agitated. Following extraction, the extracts remained at room temperature and used with 24 h of completing the extraction.

#### 2.6.3 Cytotoxicity

The cytotoxic potential of the magnesium membrane was evaluated. Extracts of the magnesium membrane, negative control, control blank, and positive control were prepared as described in section 2.6.1.

L-929 mouse fibroblasts in a semi-confluent mono layer were dosed with the extracts and incubated at 37 °C in the presence of 5% CO2 for 24 h. The cells were rinsed three times with Dulbecco's Phosphate Buffered Saline (DPBS) with Ca2+ and Mg2+ (reference D8662, Sigma-Aldrich) before fresh culture medium was added to the cells. The cells were then incubated with a cell proliferation assay (CellTiter 96® AQueous Non-Radioactive Cell Proliferation Assay with MTS and PMS, reference G5430, Promega) (MTS-PMS) solution before optical density measurements were made with a microplate reader (Tecan, Sunrise, Magellan Standard Version 6.6 software) using a wavelength of 492 nm. The percent viability was determined using the control blank as a reference. If cell viability would have dropped below 70% of the control blank, a cytotoxic potential would have been considered.

#### 2.6.4 Sensitizing

The magnesium membrane was evaluated for its potential to cause delayed dermal contact sensitization in guinea pigs (young adult, males, *Cavia porcellus*, Dunkin Hartley). This breed of guinea pig has been historically used for sensitization studies as they are believed to be the most sensitive animal model for this type of study. SC Polar and SO apolar extracts (see section 2.6.2) were intradermally injected (Induction I) and 6 days later topically applied (Induction II) to 10 test guinea pigs per extract in an attempt to induce delayed sensitization. The extraction vehicles (0.9% saline solution and the sesame oil) without extracts were similarly injected and topically applied to five control guinea pigs (per vehicle). Following a recovery period of 14 days, the challenge was applied, consisting of cotton disks saturated with the test article extract or extraction vehicle, and compressed to the trunk of the animal for 24 ± 2 h. After observational periods of 24 ± 2 h and 48 ± 2 h, each animal was scored according to the Magnusson and Kligman Scale [50].

#### 2.6.5 Irritation or intracutaneous reactivity

The magnesium membrane was evaluated for its potential to cause irritation following intracutaneous injections in rabbits (young adult, males, *Oryctolagus cuniculus*, New Zealand White). Rabbits have been historically used to evaluate biomaterial extracts and are stated in the requirements of the current ISO test standard. SC Polar and SO apolar extracts of the magnesium membrane were prepared (see section 2.6.2). A 0.2 mL dose of the appropriate test article extract was injected intracutaneously into five separate sites on the back-left side of three rabbits. Similarly, the extract vehicle alone (control blank) was injected on the back-right side of each rabbit. The injection sites were observed immediately after injection. Observations for erythema and edema were conducted at 24, 48 and 72 h after injection and scored according to the severity of the reaction. Acute systemic toxicity.

The magnesium membrane was evaluated for acute systemic toxicity in mice (Female, *Mus musculus*, OF1 Ico (IOPS Caw)). Mice have historically been used to evaluate potential toxicity and are also specified in the current ISO test standard. WFI and SO extracts of the magnesium membrane were prepared (see section 2.6.2). Five mice were given an intraperitoneal injection with a single dose (50 mL per kg of body weight) of the appropriate test article extract. Similarly, a separate group of five mice were dosed with corresponding extraction vehicle alone (blank control). The mice were observed for signs of systemic toxicity immediately after injection and at 4, 24, 48 and 72 h after injection. Body weights were recorded prior to dosing and at 24, 48 and 72 h after injection.

#### 2.6.6 Pyrogenicity

The magnesium membrane was evaluated for the potential to induce a pyrogenic response in rabbits (males, *Oryctolagus cuniculus*, New Zealand White). A rabbit model was used for this test due to the requirements of European Pharmacopoeia, 9th edition, 2016, which is a reference work for the quality control of medicines and is the basis of this test protocol. WFI extracts of the membrane were prepared (see section 2.6.2). Three rabbits received a single dose (10.0 mL per kg of body weight) of the extract. Following the injection, the rabbits had their rectal temperature measured every 30 min over a 3 h period. The sum of temperature variations of the three rabbits are used to determine pyrogenicity. A combined temperature increase of ≤1.15 °C is indicative of an absence of a pyrogen; an increase between 1.15 and 2.65 °C requires a continuation of the test; and an increase >2.65 °C is indicative of the presence of a pyrogen.

#### 2.6.7 Genotoxicity: mouse lymphoma assay

A Mouse Lymphoma Assay was performed to test for genotoxicity. The Mouse Lymphoma Assay was conducted to evaluate the mutagenic potential of the test article extracts. Using the mouse lymphoma forward mutation assay procedures, mouse lymphoma cells (L5178Y/TK^+/-^ cell line) were exposed to the extracts for a 4 h treatment in the presence and absence of metabolic activation, as well as a 24 h treatment in the absence of metabolic activation.

The test article was extracted in RPMI-1640 serum-free Cell Culture Medium (RPMI_0_) (reference R7638, Sigma-Aldrich) and Dimethyl Sulfoxide (DMSO) (reference D1435, Sigma-Aldrich). The RPMI_0_ extract solution was supplemented with 1% (v/v) l-glutamine (CAS No. 56-85-9), 1.8% (v/v) sodium pyruvate (CAS No. 113-24-6) and 2% (v/v) penicillin-streptomycin (reference P4458, Sigma-Aldrich) and 0.5% (v/v) poloxamer 188 (CAS No. 9003-11-6). Using an extraction ratio of 6 cm^2^ membrane surface area to 1 mL of extraction media, the magnesium membrane was submerged in the extraction media for 72 h and maintained at a temperature of 37 °C for RPMI_0_ and 50 °C for the DMSO. During extraction, the extracts were continuously agitated on a tube roller using 50 revolutions per minute.

Extracts tested at 8 concentrations of 100%, 50%, 25%, 12.5%, 6.25%, 3.13%, 1.56%, and 0.78% (v/v). The RPMI_0_ extract was supplemented with 5% serum prior to the 4 h and 24 h assessments. The DMSO extract was diluted to a final concentration of 1.0% with RPMI_5_ (RPMI-1640 Cell Culture Medium supplemented with 5% horse serum (reference H1138, Sigma-Aldrich), 1% (v/v) l-glutamine, 1.8% (v/v) sodium pyruvate and 2% (v/v) penicillin-streptomycin and 0.5% (v/v) poloxamer 188) for the 4 h and 24 h assessments.

The magnesium extracts were compared to blank controls (extract vehicle alone) and positive controls. Positive controls of Methyl Methanesulfonate (MMS) (CAS No. 66-27-3) and Cyclophosphamide (CP) (CAS No. 6055-19-2) were used. At the 4 h treatment, MMS was used at a final concentration of 12 and 14 μg/mL, and CP was used as a final concentration of 3 and 3.5 μg/mL. For the 24 h treatment, only MMS was used at a final concentration of 3 and 3.5 μg/mL.

#### 2.6.8 Genotoxicity: bacterial reverse mutation study

A Bacterial Reverse Mutation Study was performed to test for genotoxicity. The bacterial reverse mutation standard plate incorporation study was conducted to evaluate whether extracts of the test article or their metabolites would cause mutagenic changes in *Salmonella typhimurium* tester strains TA98, TA100, TA1535, TA1537 and *Escherichia coli* tester strain WP2*uvr*A (strains purchased from Trinova Biochem, Moltox) in the presence and absence of mammalian metabolic activation. Bacterial reverse mutation tests have been widely used for the determination of mutagenic and potential carcinogenic hazards.

Extract vehicles used were Sodium Chloride (SC) (Reference 600019, Aguettant/Lavoisier) and DMSO using the protocol described in 2.6.2. Tubes containing molten top gar were inoculated with culture from one of the five tester strains, along with the test article extracts with doses of 100 μL/plates for 100%, 50%, 25%, 12.5%, 6.25% and 3.13% (v/v) extracts. An aliquot of phosphate buffer or rat liver S9 Mixture (reference 11-01L, Trinova Biochem, Moltox) providing metabolic activation was added. The mixture was poured across the triplicate plates. Parallel testing was conducted with control blanks and positive controls. The mean number of revertants for the test extract plates was compared to the mean number of revertants of the appropriate control blank plates for each of the five tester strains.

### 2.7 *In-vivo* corrosion kinetics study in Yucatan minipigs

#### 2.7.1 Experimental animals

The experimental study was performed at BRIDGE PTS, Texas, USA using twenty-two Yucatan minipigs (*Sus scrofa*), aged between 11 and 17 months. The protocol was reviewed and approved by the Testing Facility's Institutional Animal Care and Use Committee (IACUC). The Testing Facility is accredited by the Association for Assessment and Accreditation of Laboratory Animal Care (AAALAC) and has received its Domestic Assurance certification OLAW: # A4672-01.

The pigs were anesthetized by intramuscular injection of Atropine (0.05 mg/kg) followed by Xylazine (2.7 mg/kg) and Tiletamine-Zolazepam (Tilzolan; 7.0 mg/kg, IM) and mask inhalation of 0.5–5% Isoflurane mixed with oxygen. Four pigs were assigned to each timepoint of the study (1 week ±3 days, 2 weeks ±3 days, 4 weeks ±3 days, 8 weeks ±3 days, 16 weeks ±3 days), with 2 spares.

#### 2.7.2 Surgery

On each animal, the surgeon created a 6 cm long incision approximately 2 cm below the teeth (beginning at the first premolar (PM1)) on the left and right side of the mandible. A full subperiosteal-gingival flap was raised by carefully lifting the periosteum from the underlying bone using an elevator or similar instrument.

8 mm diameter magnesium membrane disks were created using a biopsy punch prior to application. Six magnesium membrane samples were individually fixed into position using a singular titanium screw (1.5 mm × 3 mm ProFix titanium screws, Osteogenics) into each minipig. The flaps were closed hermetically using traditional surgical techniques and with interrupted suturing.

Mandibles were removed upon termination. Using X-rays taken of the mandibles, the implants were located and extracted using a core drill with a 10 mm inner diameter. The extracted samples containing the membrane and boney material were stored in plastic containers with an inner diameter of 10 mm and frozen at −80 °C.

#### 2.7.3 Micro computed tomography

To evaluate changes to the membrane structure, samples from each timepoint were imaged using synchrotron-radiation based microtomography (SRμCT). SRμCT has been extensively exploited for 3D investigation of magnesium implant degradation [51], and the μCT setups at the ESRF are well established for the investigation of bone [[52], [53], [54]].

3D SRμCT datasets were acquired using the μCT setups called beamline ID19 at the ESRF in Grenoble. The energy of the beam (pink-beam) was set to 43 keV and pixel size was set to 2.2 μm. The number of projections per tomographic scan was 3999. To cover the full screw inside the resulting field of view, 1-3 scans were collected for each sample at different z-positions with ∼20% overlap between individual scans. Reconstruction was performed using Paganin's method in combination with the conventional filtered back projection algorithm applying a delta/beta ratio of 350. Reconstructed data were stored in units of refractive indices in units of 2π/λ, with λ being the wavelength of the X-ray beam, referred to as grey value data, stored in 32bit floating values.

### *2.8* *In-vivo* performance study in Beagle dogs

The overall purpose of this study was to evaluate the safety and efficacy of the NovaMag® magnesium membrane compared to the control collagen membrane (Bio-Gide®, Geistlich AG), placed over bone defects filled with bone substitute material (BioOss, Geistlich AG) in healed extraction sites, and fixed with titanium screws using a canine mandibular defect model at an early timepoint (1 week), intermediate timepoint (8 weeks), and late timepoints (16 weeks and 52 weeks) post-implantation.

#### 2.8.1 Animal model

The dog model represents a fully functional *in vivo* anatomical model for bone healing evaluation following defect creation and bone remodeling [[55], [56], [57], [58]]. The mandibular defect model allows to assess and compare the local tissue effects and the performance of the membranes. Adult dogs have a similar bone density to humans and canine bones are representative of the implantation of human implants and prostheses. Canine bone tissue exhibits similar mechanical properties, morphological structures and healing capacity to human bone. In addition, dog bones are also large enough to allow multiple experimental procedures [[55], [56], [57], [58]]. As this study evaluated the healing process following a surgery, in-vitro or computer-generated models cannot be used.

#### 2.8.2 Surgical procedure

**Fig. 3:**
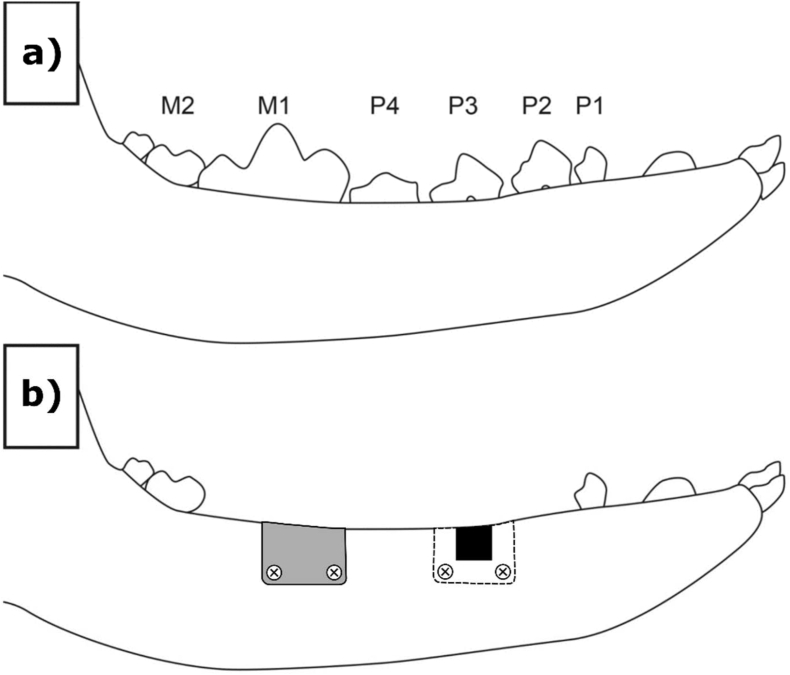
Approximate positions of the defect/implant sites used for the Beagle dog *in vivo* study. a) In a preparatory surgery, four teeth between the mandibular second premolar to the first molar (PM2 to M1) on each side of the lower jaw and the corresponding teeth of the upper jaw were surgically extracted. b) After a healing period of 12 ± 2 weeks, two independent bone defects were created on each side of the lower jaw. The defects were filled with bone substitute material and covered with a magnesium or collagen membrane fixed with 4 titanium screws (2 on buccal and 2 on lingual side). The left side defect in b) shows the positioning of the magnesium membrane secured with fixation screws and the right side defect demonstrates the position of the defect under the membrane.

In 20 beagles the preparatory phase (demonstrated in Fig. 3a) was surgical extraction of four (4) teeth from the mandibular second premolar to the first molar (PM2 to M1) on each side of the lower jaw (mandible) and the corresponding teeth of the upper jaw (maxilla) were extracted, followed by wound closure of the upper jaw only. A healing period of 12 ± 2 weeks followed with suture removal at about 2 ± 1 week's post-extractions.

In the experimental phase (demonstrated in Fig. 3b), a second surgery was performed according to the procedure as given in Fig. 1. Two independent bone defects (5 mm diameter, 5 mm depth, 10 mm apart from each other and from the next tooth, no cortical wall defects) were created from the mandibula crest on each side of the lower jaw only, (N = 4). The defects were filled with bone substitute material (BioOss, Geistlich AG) and covered with either the magnesium membrane or the collagen membrane (Bio-Gide®, Geistlich AG). Each membrane was fixed with 4 titanium screws, ProFix Screws, (2 on the buccal and 2 on the lingual side). Each animal received a total of 4 defect sites implanted with a total of 4 membranes. Suture removal was performed at about 2 ± 1 week post-implantation.

#### 2.8.3 Histology processing

Non-decalcified histology of the tissues surrounding the defect sites was performed. Implanted bone defects were separated individually with an appropriate bandsaw. Individual blocks containing the implant and the surrounding soft and hard tissues were embedded in methylmethacrylate (MMA) resin. The blocks were cut in a bucco-lingual plane using a diamond saw. One central area was harvested from each block and then two sections were ground from this area to a thickness of about 60 μm. The histologic slides were stained with Goldner's Trichrome. The Goldner's Trichrome stained sections were digitally captured to obtain whole-section images, which were then analyzed using Image-Pro Premier 9.2 or higher software.

Histopathological evaluation of tissue response to the magnesium membrane and collagen membrane was completed by board-certified veterinary pathologist, via light microscopy. All slides were evaluated to assess the local tissue healing response. Macroscopic observations were provided by AccelLAB Inc., 1635 Lionel-Bertrand Blvd., Boisbriand (Quebec) J7H 1N8, Canada and External Pathology Site, Vet Path Services, Inc (VPS), 6450 Castle Drive Mason, OH 45040 USA for evaluation and microscopic correlation. The sections were analyzed and graded according to cell type and responses. GT slides were generated from tissue in the center of the defect, to assess the inflammatory response, necrosis, neovascularization, fibrosis, fatty infiltrate, tissue degeneration, the new bone growth, evidence of membrane, soft tissue infiltration, and void space parameters (Table 1). The findings in the histopathological analysis were graded according to the following scale: 0 = Absent; 1 = Minimal; 2 = Mild; 3 = Moderate; 4 = Marked.

**Table 1:** Histological grading according to the cell type and tissue response.

Response	Score *(phf = per high powered (x400) field)				
	0	1	2	3	4
Polymorphonuclear cells	0	Rare, 1-5/phf*	6-10/phf	Heavy infiltrate	Packed
Lymphocytes	0	Rare, 1-5/phf	6-10/phf	Heavy infiltrate	Packed
Plasma cells	0	Rare, 1-5/phf	6-10/phf	Heavy infiltrate	Packed
Macrophages	0	Rare, 1-5/phf	6-10/phf	Heavy infiltrate	Packed
Giant cells	0	Rare, 1-2/phf	3-5/phf	Heavy infiltrate	Packed
Necrosis	0	Minimal	Mild	Moderate	Marked
Fibrinous exudates	0	Minimal	Mild	Moderate	Marked
Tissue degeneration	0	Minimal	Mild	Moderate	Marked
Neovascularization	0	Minimal capillary proliferation focal, 1-3 buds	Groups of 4–7 capillaries with supporting fibroblastic structures	Broad band of capillaries with supporting structures	Extensive band of capillaries with supporting fibroblastic structures
Fibrocytes/fibroconnective tissue, fibrosis	0	Narrow band	Moderately thick band	Thick band	Extensive band
Fatty infiltrate	0	Minimal amount of fat associated with fibrosis	Several layers of fat and fibrosis	Elongated and broad accumulation of fat cells about the implant site	Extensive fat surrounding the implant
**Additional Parameters**					
Overall Inflammation, Inflammation within Gingiva (away from membrane), Inflammation Associated with membrane/Membrane Area, Inflammation Associated with Bone Filler, New Bone Growth, Soft Tissue Infiltration, Hemorrhage, Amount of Void/Empty Space	Absent	Minimal	Mild	Moderate	Marked

The GT stained slides were also used to obtain histomorphometric data of interest. Histology measurements targeted evaluation of bone regeneration by measuring the new bone formation and soft tissue infiltration, within the original defect area. For each group, the mean measurements and standard deviation were calculated for: Percentage of bone area (BA%); Percentage of bone substitute material area (IA%), which included bone substitute inside and outside bone area; Percentage of soft tissue area (ST%; i.e. the percentage of soft tissue, including fibrosis and marrow, within the original defect area); and Percentage of void area (VA%). The region of interest was defined by the original defect area, with the upper limit of the region of interest being the implanted membrane.

#### 2.8.4 Statistical analysis

Equal variance and normality tests were performed. When equal variance and normality were observed, then a *t*-test was used to test for differences in continuous variables between study groups. When either equal variance test or normality test failed a Mann-Whitney Rank Sum Test was performed. A value of p < 0.05 was considered statistically significant.

## 3 Results

### 3.1 Material

Results of the ICP-MS, ICP-OES and AAS analysis are shown in Table 2. Of the trace elements present in the magnesium metal, manganese (Mn) with 237 ppm and aluminum (Al) with 131.7 ppm account for the largest proportion of foreign elements. Iron (Fe), zinc (Zn), silicon (Si) and calcium (Ca) are also detectible at low concentrations above the detection limits. All of the other tested elements are below the respective detection limit of the used analytical methods.

**Table 2:** Results of the Analysis regarding the chemical composition of raw material.

Analyzed chemical element (test method)	Mean content of chemical element in ppm (mg/kg) of three raw material batches
Fe (ICP-MS)	39,4
Al (ICP-MS)	131,7
Be (ICP-MS)	<10
Cu (ICP-MS)	<10
Ni (ICP-MS)	<10
Cr (ICP-MS)	<10
Mn (ICP-MS)	237,0
Zn (ICP-MS)	37,2
Ca (ICP-MS)	10,2
V (ICP-MS)	<10
As (ICP-OES)	<30
Cd (ICP-OES)	<30
Hg (ICP-OES)	<30
Pb (ICP-OES)	<30
Si (ICP-OES)	30,5
Na (AAS)	<10

#### 3.1.1 Material microstructure

**Fig. 4:**
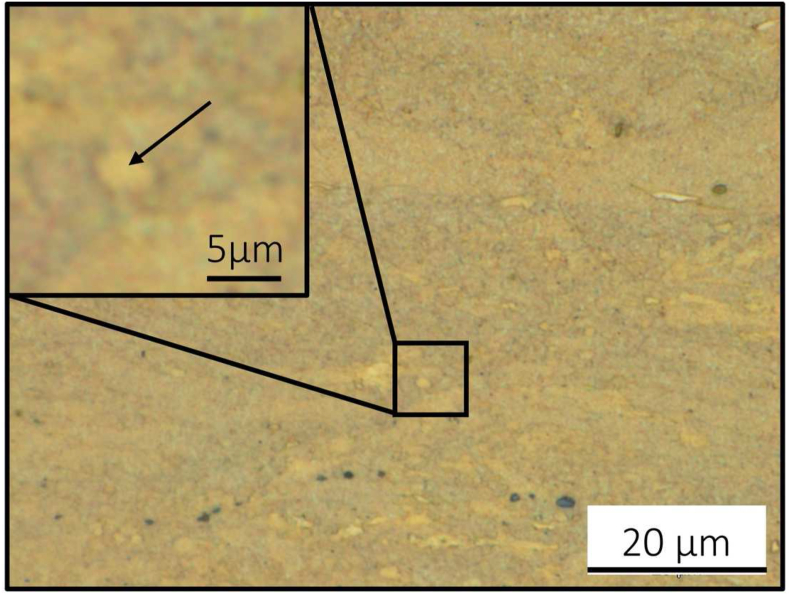
Microstructure of pure Mg membrane (99.95 wt%). Not fully recrystallized structure with an almost homogenous distribution of grains in the range of 1–10 μm (arrow in enlarged insert). Black areas are artifacts from sample preparation.

The microstructure of the pure Mg membrane (99.95 wt%) was not fully recrystallized with an almost homogenous distribution of grains in the range of 1–10 μm (arrow in enlarged insert, Fig. 4). The preparation of the metallurgical samples and the analysis were challenging due to the fast oxidation of the high-purity magnesium surface leading to artifacts which appeared as black areas in light microscopy (Fig. 4).

### 3.2 Mechanical testing

**Fig. 5:**
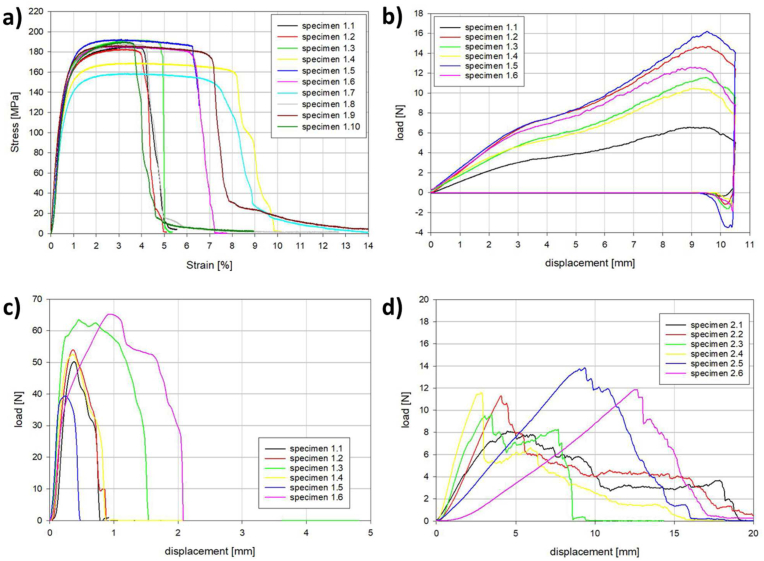
Mechanical test data for: a) Tensile testing of the magnesium membrane; b) Bend test of the magnesium membrane; and the Ultimate Load at Anchor Point for c) the magnesium membrane and d) a collagen membrane.

The mechanical properties of the magnesium membrane were characterized using a series of mechanical test, the results of which are shown in Table 3. A static tensile load test was used to determine the resistance of the magnesium membrane to tearing (n = 10). The yield point was reached with a tensile stress of 84.1 ± 7.4 MPa creating an elongation of 0.3 ± 0.0% (Fig. 5a). The maximum tensile stress was 183.0 ± 10.7 MPa with an elongation of 3.3 ± 0.3%. Although all of the samples failed by fracture during the test, the percentage elongation at fracture could not be determined because there was no distinct point when breakage occurred. The elastic modulus for the magnesium membrane was 34.2 ± 3.4 GPa.

**Table 3:** Magnesium membrane mechanical properties.

Tensile Test	
Tensile Stress at Yield	84.1 ± 7.4 MPa
Percentage Elongation at Yield	0.3 ± 0%
Maximum Tensile Stress	183 ± 10.7 MPa
Elongation at Maximum Tensile Stress	3.3 ± 0.3%
E-Modulus	34.2 ± 3.4 GPa
**Bending Test**	
Maximum Load	12 ± 3.4 N
Displacement at Maximum Load	9.3 ± 0.3 mm
Bending Stiffness	1.8 ± 0.5 N/mm
**Ultimate Load at Anchor Point**	
Magnesium Membrane	54.1 ± 9.4 N
Collagen Membrane	11.1 ± 2.0 N

A static bending test determined the ability of the magnesium membrane to undergo plastic deformation and the integrity of the surface structure after deformation. The membranes (n = 6) withstood a maximum load of 12.0 ± 3.4 N, which was associated with a 9.3 ± 0.3 mm displacement (Fig. 5b). Overall, a bending stiffness of 1.8 ± 0.5 N mm^−1^ was calculated. Once bending occurred, it resulted in a permanent deformation of the magnesium membranes. A visual inspection determined that the deformation of the magnesium membrane did not cause damage to its surface.

The magnesium membrane was tested for its ability to resist tearing at its point of fixation to provide an assessment of its fixation stability. For all of the magnesium membrane samples (n = 6), the fixation screw that was anchoring the magnesium membrane was pulled out of the block with an average tensile load of 54.1 ± 9.4 N before the membrane could tear or break (Fig. 5c). Therefore, it was the fixation stability of the fixation screw that failed before failure of the membrane could occur. For the collagen membrane, the fixation screw maintained its position within the synthetic bone block and the membrane tore under a tensile load of 11.1 ± 2.0 N.

### 3.3 In-vitro corrosion

**Fig. 6:**
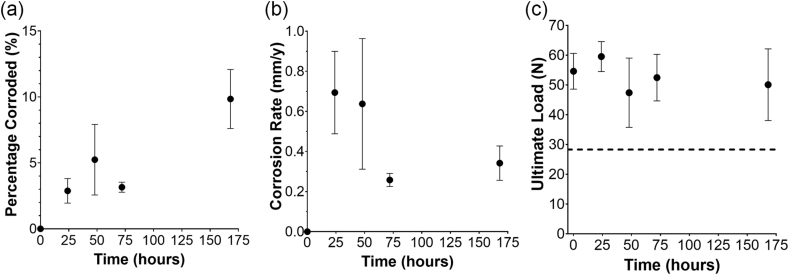
In vitro immersion corrosion test results: a) the percentage of the membrane corroded in comparison to its initial weight; b) the respective corrosion rate calculated for each time point; and c) is the results of the “small punch test”, with the result of the undegraded collagen membrane shown as a horizontal dashed line at 28.3 ± 1.6 N.

During the immersion corrosion test *in vitro*, the magnesium membrane initially lost about 8 mg per day (n = 5 per time point). The corrosion rate decreased over the first 3 days (Fig. 6), although by day 7, the average corrosion rate had slightly increased from that of day 3 (non-significantly). By the end of the test, the membranes experienced an average mass loss of 4 mg per 24 h. Within a period of 1 week during the *in vitro* corrosion study, the magnesium membrane had lost approximately 10% of its initial mass (Fig. 6).

The ability of the membrane to resist puncture after different periods of degradation was evaluated with the punch test (n = 5 per time point). A time point of 0 h was used to compare the magnesium membrane's initial resistance to that of a collagen membrane before it was degraded. At this time point, the magnesium membrane had an ultimate loading force of 54.6 ± 6.0 N, which was almost double that of the collagen membrane that had an ultimate loading force of 28.3 ± 1.6 N. Over the 7 day period, the magnesium membrane retained most of its strength, with average ultimate load values between 47.4 N and 59.5 N (Fig. 6c). The ultimate displacement of the magnesium membrane remained similar between each time point (0.5–0.9 mm), and was approximately half of that of the collagen membrane (1.4–1.7 mm).

### 3.4 Biological safety assessment

The biological safety of the magnesium membrane was evaluated using cytotoxicity, sensitization, systemic toxicity, pyrogenicity and genotoxicity tests.

Cytotoxicity was evaluated via an *in-vitro* study by measuring the metabolic activity and the proliferation of L929 mouse fibroblast cells in contact with extracts from the magnesium membrane, the results of which are presented in Table 4 The extract presented a cytotoxic potential to L-929 cells at 100% (full strength) and at dilutions of 50%, 25% and 12.5% (v/v). At dilutions of 6.25% and 3.13% (v/v), the extracts showed no cytotoxic potential to L-929 cells. A maximum 10 times dilution of the extract is acceptable to pass the cytocompatibility testing according to ISO10993-5, as the concentration (osmolality) of the dissolved magnesium within the *in-vitro* test does not represent the real concentration of the degradation products *in-vivo*. *In-vivo*, the osmolality changes are buffered immediately. Therefore, the magnesium membrane passed the cytocompatibility test using a 5 times dilution (6.25% concentration).

**Table 4:** Cytotoxicity of magnesium membrane extracts at different concentrations.

Extract Concentration	Percent Viability	Cytotoxic Potential
100%	11.3%^a^	Cytotoxic
50%	1.2%	Cytotoxic
25%	1.4%	Cytotoxic
12.50%	57.1%	Cytotoxic
6.25%	74.3%	Not Cytotoxic
3.13%	78.3%	Not Cytotoxic

Extracts from the magnesium membrane were evaluated for sensitization reactions using an albino guinea pig model (Magnusson-Kligman test). Under the conditions of the study, neither the polar nor apolar (n = 5 per group) extracts caused delayed sensitization, thus the magnesium membrane is not considered to be a sensitizer.

a Overestimation due to presence of particulates in one well tested even after several cell rinses.

For irritation and intracutaneous reactivity experiments in rabbits, both the polar and apolar extracts did not cause any immediate reactions (n = 15; 5 sites in 3 rabbits). When compared to controls for observations of erythema and edema, both extracts produced results equivalent to the controls. Therefore, the magnesium membrane extract did not cause irritation and intracutaneous reactivity.

During the systemic toxicity experiments in mice, there was no mortality or evidence of systemic toxicity from the WFI and SO extract injections (n = 5 per group). Therefore, the magnesium membrane does not cause acute systemic toxicity.

Pyrogenicity tests in rabbits did not demonstrate any pyrogenic effects (n = 3) (Table 5). Over the course of the 3 h test period, the combined increase in rabbit body temperature did not exceed 1.15 °C, which is the pre-set condition of the test when using 3 rabbits. Therefore, the magnesium membrane extract was demonstrated to have a non-pyrogenic effect in rabbits.

**Table 5:** Pyrogenicity results for extracts of the magnesium membrane in rabbits.

Rabbit	Temperature (°C)							
	Av. before injection	After Injection						Temperature Rise
		0.5 h	1.0 h	1.5 h	2.0 h	2.5 h	3.0 h	
1	39.04	39.4	39.29	39.29	39.26	39.33	39.36	0.36
2	38.98	39.2	39.22	39.18	39.17	39.14	39.09	0.25
3	39.19	39.34	39.36	39.36	39.34	39.29	39.41	0.28
Total								0.89

For the mouse lymphoma assay testing for genotoxicity, both the RPMI_0_ and DMSO extracts with concentrations between 0.78 and 100% (v/v) showed no mutagenicity. Moreover, no mutagenic or genotoxic activity was caused by the magnesium membrane extract used in the bacterial reverse mutation study. Therefore, the magnesium membrane is not considered genotoxic.

### *3.5* *In-vivo* corrosion kinetics study in Yucatan minipigs

The *in-vivo* corrosion kinetics of the magnesium membrane were evaluated using a synchrotron. The high sensitivity and resolution of the synchrotron μCT was able to depict the metallic magnesium by distinguishing it from the corroded magnesium and bone tissue, even when presented with similar densities. This enabled a qualitative description of the changes occurring during the corrosion of the magnesium membrane. However, as the membrane progressively corroded, it became more challenging to differentiate from the surrounding tissue.

Due to several instances where the titanium fixation screw was lost after implantation, locating the magnesium membrane was not always possible. In other instances, the titanium pin remained without signs of the magnesium membrane. In these instances, it could not be determined if the membrane had been lost or completely corroded. Therefore, at each time point, a different number for samples were imaged with SRμCT: at 2 weeks, 17 samples; 4 weeks, 14 samples; 8 weeks, 8 samples; and at 16 weeks, 5 samples.

**Fig. 7:**
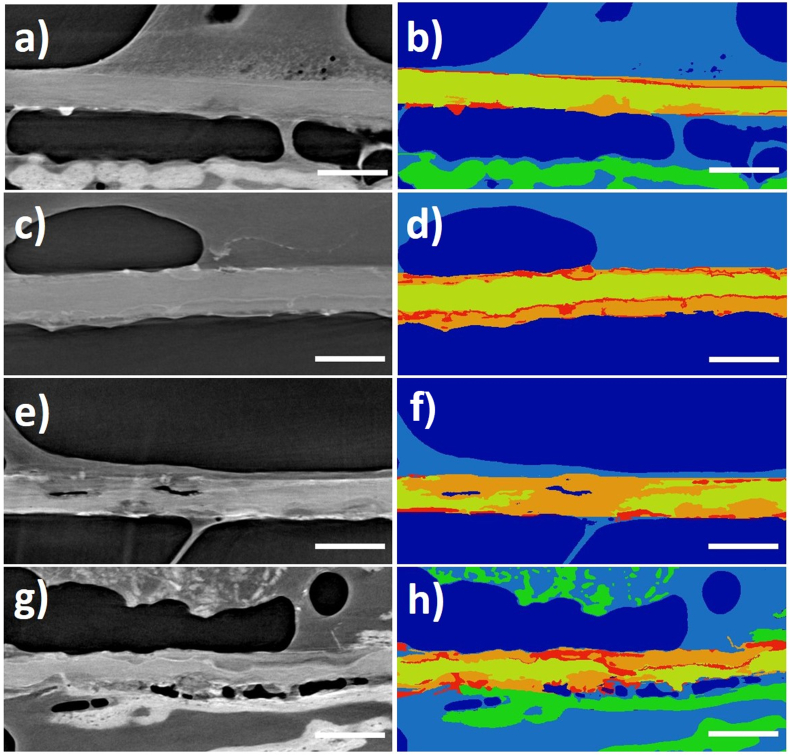
SRμCT images demonstrating the corrosion kinetics of the magnesium membrane when implanted into Yucatan minipigs after: 1 week (a, b), 2 weeks (c, d), 4 weeks (e, f), and 8 weeks (g, h). The scale bar in each image represents 250 μm. Grey scale images (a, c, e, g) and false colored images (b, d, f, h) are shown to emphasize magnesium membrane location within the surrounding tissue and display magnesium metal corrosion products. In the colorized images: Soft tissue (light blue) and mineralized bone tissue (green) adhere to the surface of the membrane. The magnesium metal (yellow) can be seen to gradually corrode into magnesium salts (orange and red). (For interpretation of the references to color in this figure legend, the reader is referred to the Web version of this article.)

Images of the segmented magnesium membranes are shown in Fig. 7. As the membrane corroded, the denser material of the corrosion by-products appears as corrosion fronts on the membrane. The corrosion fronts are potentially composed of magnesium-calcium -phosphate salts that prevent the further corrosion of the magnesium until they fracture and break off [59]. This then promotes the next phase of local corrosion, thus multiple corrosion fronts can be found at each timepoint of the study. At 1, 2 and 4 weeks, the magnesium salts retain the original shape and position of the magnesium metal until they themselves are resorbed, preserving a separation of the soft and hard tissues.

At the 8 week timepoint, the magnesium had severely corroded and therefore could not always be segmented. In these samples, the potential loss of the membrane was disregarded due to the presence of corrosion by-products still present in the surrounding tissue. As the membrane corroded, new bone formation was evident within a close proximity to the membrane lower surface. Over the course of the study, the new bone matured into a lamella structure. Soft tissue attached to the top surface of the membrane, however with the presence of the hydrogen gas pockets, not all of the membrane's upper surface was covered in tissue.

### 3.6 *In-vivo* performance study in Beagle dogs

#### 3.6.1 Surgical procedure

By following the implantation procedure outlined in Fig. 1, all magnesium membranes were successfully implanted without complications. There were no instances of morbidity or mortality that required replacement animals to be used.

#### 3.6.2 Histology

Within the dental defect sites, overall inflammation was slightly greater in the magnesium membrane group at 1 week, similar in both groups at 8 weeks, and lower in the magnesium group at 16 weeks (Table 6). Inflammation within the gingiva overlying the membranes remained as minimal (an approximate score of 1) throughout the investigation. At every time point, the inflammation was mostly associated with the membrane due to the bioresorption process, with variable amounts of inflammation occurring either within the gingiva away from the membrane site, or surrounding the bone filler within the defect site. At 52 weeks, inflammation was generally low in both treatment groups, with a slight increase in overall inflammation in the magnesium membrane group due to one test site having a score of 2 for inflammation (macrophages only), with evidence of continued remodeling occurring in the superficial aspect of that site. The other three sites in the magnesium membrane group at 52 weeks had scores of 1, similar to the appearance of the collagen membrane sites. The inflammation was typically composed mostly of neutrophils with few macrophages at 1 week, which transitions into mostly macrophages (located within the tissue in the area of the membrane, within the gingiva away from the membrane, and surrounding the bone filler material) and occasional multinucleated giant cells (surrounding bone filler material) at 8 weeks. There was a similar trend between the 8 and 16 week samples in both treatment groups. At 52 weeks, only macrophages were noted (mostly within the membrane area or gingiva away from the membrane), with one magnesium treatment site demonstrating minimal (score 1) inflammation associated with the bone filler due to continued remodeling in the superficial aspect of that defect site.

**Table 6:** Histopathological summary of inflammatory response.

Parameter	Week1		Week 8		Week 16		Week 52	
	Mg	Col.	Mg	Col.	Mg	Col.	Mg	Col.
	(n = 12)	(n = 12)	(n = 12)	(n = 12)	(n = 12)	(n = 12)	(n = 4)	(n = 4)
Overall Inflammation	2.92 ± 0.5	2.67 ± 0.6	1.50 ± 0.6	1.50 ± 0.6	1.33 ± 0.6	1.58 ± 0.8	1.25 ± 0.4	1.00 ± 0.0
Inflammation within Gingiva (away from membrane)	1.00 ± 0.4	0.92 ± 0.3	1.00 ± 0.0	1.08 ± 0.3	1.17 ± 0.6	1.08 ± 0.3	1.00 ± 0.0	1.00 ± 0.0
Inflammation Associated with Membrane/Membrane Area	2.92 ± 0.5	2.58 ± 0.6	1.50 ± 0.6	1.50 ± 0.6	1.17 ± 0.4	1.58 ± 0.8	1.25 ± 0.4	1.00 ± 0.0
Inflammation Associated with Bone Filler	1.58 ± 0.5	1.17 ± 0.6	1.00 ± 0.0	1.00 ± 0.0	1.08 ± 0.3	1.00 ± 0.0	0.25 ± 0.4	0.00 ± 0.0

**Fig. 8:**
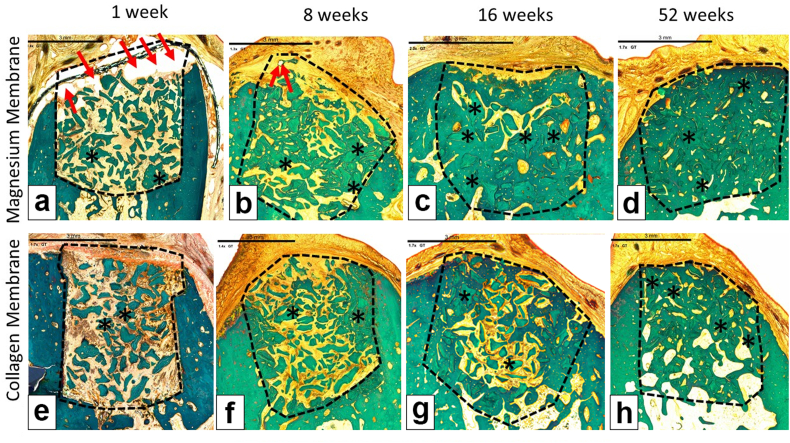
Representable scanned Goldner's Trichrome histology images of GBR performance study on beagles. Dotted Line = edges of the defect site; Asterisks (*) = particles of bone filler material within the defect site; Red Arrow = void/cavity/gas space; (a), (b), (c) and (d) are presenting the magnesium membrane where we can see that is degrading/reabsorbing over time, and by 8 weeks (b), only small residual particles of the magnesium membrane are left, surrounded by new bone and little part of void space. At 16 weeks (c) and 52 weeks (d) the magnesium membrane is completely absorbed and replaced by new bone. (e), (f), (g) and (h) are presenting a collagen membrane at all time points; 1 week (e), 8 weeks (f), 16 weeks (g) and 52 weeks (h). In each image, the scale bar represents 3 mm. (For interpretation of the references to color in this figure legend, the reader is referred to the Web version of this article.)

For biocompatibility, neovascularization was similar across both treatment groups at all time points. New bone growth was expectedly low in both treatment groups at 1 week, with scores of 1 (minimal) in each site due to the early time point. New bone growth increased significantly by week 8, with similar scores noted for both treatment groups, and at 16 weeks the average new bone growth scores were greater in the magnesium membrane group (2.92) compared to the collagen membrane group (2.50). By 52 weeks, the new bone growth was evident (score 4) in all sites in both treatment groups, demonstrating reconstruction of the alveolar bone within the defect site (Fig. 8). The average score for soft tissue infiltration was slightly greater in the collagen membrane group compared to the magnesium membrane group at 1 week; this was due, in part, to the presence of void spaces occurring most commonly in the superficial defect site surrounding the membrane, but also extending into the mid-defect site in some sites in the magnesium group at this early time point. The void space was presumably due to very early degradation of the magnesium membrane, with production of hydrogen gas.

**Fig. 9:**
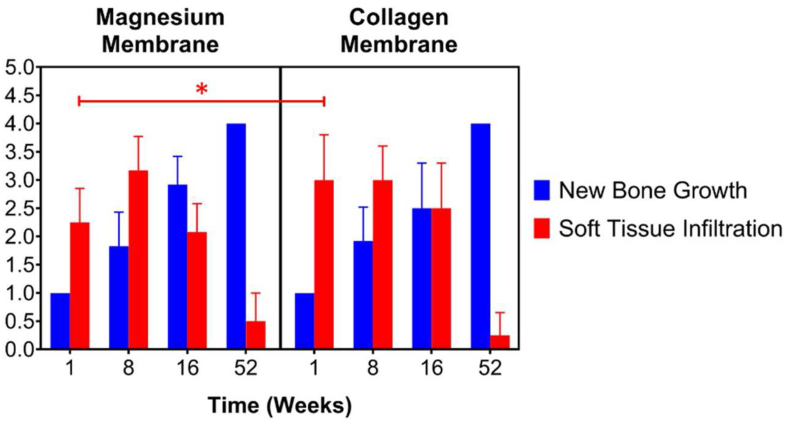
Histology measurements of new bone formation and the soft tissue infiltration. Certain measurements for New Bone Growth show no standard deviation due to uniformity of results at these specific timepoints.

Soft tissue infiltration decreased from 1 week to 16 weeks, with a lower average score (2.08) occurring in the magnesium membrane group at 16 weeks compared to the collagen membrane group (2.50). The void space decreased significantly from 1 week (average score 3.08) to 8 weeks (average score 0.67), with no evidence of void space noted at 16 weeks in the magnesium membrane group. At 52 weeks, the soft tissue infiltration scores were quite low, with the magnesium group having a slightly greater average score (0.50) compared to the collagen group (0.25) (Fig. 9).

The overall irritancy/reactivity score for the magnesium group was 0.50, 0.00, 0.00, and 0.50 at 1 week, 8 weeks, 16 weeks, and 52 weeks, respectively, and thus the magnesium group was deemed to be a non-irritant at all time points.

**Fig. 10:**
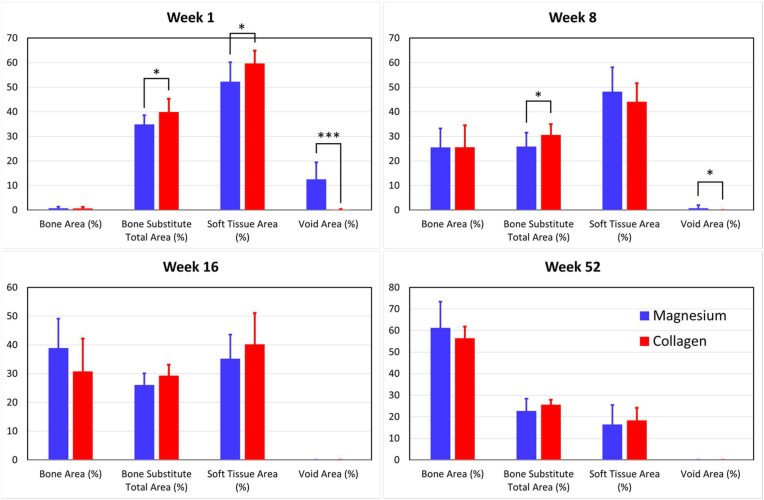
Histomorphometric measurements for the percentages of bone area, bone substitute total area, soft tissue total area and void area within the defect site of magnesium membrane and collagen membrane treatment groups.

Histomorphometric measurements of the defect site for each timepoint of the study are shown in Fig. 10. The bone area percentage increased in both groups between every timepoint, with a greater average bone area percentage occurring for the magnesium membrane group compared to the collagen membrane group at 16 and 52 weeks. However, the number of samples for each treatment group was much lower at 52 week (n = 4) compared to the other timepoints (n = 12) and thus these trends were less definitive at 52 weeks.

The bone substitute material in both groups decreased from 1 week to 8 weeks, but appeared to remain at a similar level between weeks 8 and 16. During this period, it is unclear as to the fate of this disappearing bone substitute material, as it may have been reabsorbed, or may have migrated outside the evaluation area. At both the 8 week and 16 week timepoints, the collagen membrane group had most of its bone substitute material present outside areas of new bone growth and fewer bone substitute granules surrounded by areas of new bone growth. In comparison, at 16 weeks, the magnesium membrane group had most of its bone substitute within areas of new bone growth. At 52 weeks, the vast majority of the bone substitute material in both groups was located within regions of new bone growth, with both treatment groups demonstrating evidence of full or nearly full healing of the sites.

There was a decrease in overall soft tissue area percentage in both groups over time. This occurred as the soft tissue in the defect was replaced with areas of new bone growth. The total decrease in soft tissue area percentage was similar in both treatment groups.

The overall void area percentage for the magnesium membrane group decreased from 12.37% at 1 week, to 0.72% at 8 weeks, and finally resulted in 0.00% at 16 weeks with no void area present at 52 weeks. Thus, the void area was significant early in the biodegradation and bioresorption process (i.e. at 1 week) due to hydrogen gas production, but upon full bioresorption of the magnesium membrane at 16 weeks, the void space appeared to completely resolve.

## 4 Discussion

A magnesium membrane has been developed as an alternative resorbable barrier membrane to be used in GBR surgeries. The material properties of pure magnesium could provide all the ideal characteristics for a GBR membrane whilst addressing the issues of currently used membranes, such as poor mechanical strength or the requirement for a second surgical procedure for membrane extraction. As defined by Gentile et al. and Rakhmatia et al., the ideal characteristics of a barrier membrane are: clinical manageability for fast and efficient insertion, space provision of the defect void for bone ingrowth, seclusion of the overlying epithelial and connective tissues from the void space, biocompatibility, non-immunogenicity, non-toxicity, and finally tissue integration [3,4]. Each criterion has been investigated in relation to the magnesium membrane and is presented in this paper.

Clinical manageability was confirmed during the *in vivo* performance study using a GRB model in Beagle dogs, as all of the membranes were successfully implanted.

The ability of the membrane to resist collapse into the defect void and maintain space for the ingrowth of new bone is viewed as an important aspect for producing a successful regenerative outcome [60]. This is most easily achieved by using a strong material that can resist the external pressures of the overlying soft tissue and the forces experienced during mastication [4]. The risk of collapse tends to be higher for resorbable membranes due to inferior mechanical properties [61].

The resistance of the magnesium membrane to collapse was evaluated using a static tensile strength test. Previous tensile tests performed on commercially available collagen membranes have indicated a range of maximum tensile stresses between 4.8 and 22.5 MPa [62,63], and ∼14.5 MPa for resorbable polymeric membranes [64]. However, the tensile tests are often performed under dry conditions due to the decline in collagen tensile strength when wet [63]. During their surgical placement, the membranes will become wet via contact with patient saliva and blood. The reduction in mechanical properties when wet additionally risk collagen membranes tearing during placement. In contrast, the results for the magnesium membrane demonstrated a high stability and a low elasticity. Due to its metallic structure, wetting during surgical placement will not affect its handling. During testing, the magnesium membrane measured a maximum tensile stress of 183.0 ± 10.7 MPa, which is substantially larger than that for the collagen and polymeric membranes. Therefore, the risk of collapse into the defect void, as reported for collagen [20,21] and polymeric membranes [61], could potentially be prevented by using a magnesium membrane.

Additionally, the magnesium membrane was demonstrated to have a high resistance to being punctured. Puncturing of a membrane could occur during mastication, impeding the cell occlusive barrier. During puncture tests, the resistance of the magnesium membrane to penetration was approximately double that of the comparative collagen membrane. Even after 7 days under degradative conditions, the magnesium membrane maintained a higher resistance to puncture than that of the undegraded collagen membrane (Fig. 6c).

In GBR surgeries, it is common to secure barrier membranes in place using fixation screws. The fixation of a membrane helps to prevent its dislocation as well as the transfer of micromovements to the augmentation site [6]. Micromovements are known to prevent bone formation and cause the formation of fibrous tissue [65]. By fixating the membrane there is also the potential to increase vertical bone gain [66] and more rapidly create an organized bone structure [65].

To determine the stability of the magnesium membrane at its point of fixation, hence its ability to resist micromovements and remain in position, a tear resistance test was performed using a singular anchorage point provided by a titanium screw. In the test, the resistance of the magnesium membrane to tearing was so great that the anchorage of the titanium fixation screw failed (at a tensile load of 54.2 ± 9.4 N) before the membrane could tear. In contrast, a tested collagen membrane used as a comparator, tore at a tensile load of 11.1 ± 2.0 N, demonstrating that a more secure fixation can be achieved with the magnesium membrane.

Securing the magnesium membrane using a metallic fixation screw could potentially cause a problem due to galvanic corrosion. It is widely known that titanium can increase the dissolution rate of magnesium by galvanic coupling [67]. The reason for this is the overlapping of the corrosion potential of magnesium with the potential for hydrogen evolution on the titanium surface. The degree of galvanic coupling depends on many factors, including the surface area ratio between the anode (in the present case the magnesium membrane) and the cathode (in the titanium screw), the spacing between each implant and the degree of electrical/ionic conductivity. When there is a risk of galvanic corrosion, a large ratio between the anode/cathode area is a way to mitigate detrimental effects on the material of interest. Also a good conductivity will lead to a more general form of corrosion, instead of a localized type that could be detrimental to the mechanical stability of the membrane in the vicinity of the screw.

An assessment of this effect has been made using voltammetry measurements in HBSS with varying surface area ratios between the magnesium membrane and titanium, the results of which will be published separately. To summarize these results, it has been found that a surface area ratio of 10(Mg):1(Ti) or higher does not enhanced the risk of local galvanic corrosion. This implies that a magnesium membrane of 15 × 20 mm can be safely fixated using two titanium screws (25 mm^2^ surface area, equivalent to ProFix titanium screws), a 20 × 30 mm magnesium membrane can be safely fixated using four titanium screws, and a 30 × 40 mm magnesium membrane can be safely fixated using six titanium screws. This number of screws are at or above the maximum number typically used for membrane fixation in clinical practice. A homogenous corrosive attack was confirmed by microscopic observations of the magnesium membranes which were fixated by titanium screws in HBSS up to 54 h. No enhanced local corrosion of the magnesium membrane around the titanium screws has been observed.

Biocompatibility issues with existing synthetic polymer membranes can adversely affect the healing response in patients [[68], [69], [70]]. Tatakis and Trombelli reported the use of a PLA barrier membrane in 27 patients undergoing guided tissue regeneration treatment [68]. Severe swelling and histopathological evidence of a foreign body reaction was observed in approximately half of the patients and defects treated. In some instances, this foreign body reaction has been reported to cause resorption of the bone [70,71]. Due to the long degradation time for PLA membranes, the foreign body reaction and bone resorption can occur over a prolonged period of time, with Schmitz et al. reporting reoccurring instances in one patient over a 12 month period [69]. Biocompatibility, non-immunogenicity, and non-toxicity were proven for the magnesium membrane through a series of *in-vitro* and *in-vivo* studies. The tests were selected according to ISO 10993 and performed to fulfil the standard's requirements. ISO 10993 is a series of internationally recognized standards used for evaluating the biocompatibility of medical devices.

As was demonstrated with the case report by Schmitz et al., a slow degradation rate of the membrane can have some unintended consequences [69]. Conversely, a resorption period that is too short will prevent the necessary separation of the soft and hard tissues that is required for an optimal regenerative result [72]. In the membrane performance study using a dog model, the main part of the magnesium membrane corroded after 8 weeks. During this period, the membrane retained the augmentation material within the bone defect. This is shown, as none of the bone granules can be observed outside the bone defect at any timepoint of the study (Fig. 8). Moreover, the augmentation does not appear to be misshapen or indented, thus proving the mechanical protective function of the magnesium membrane.

An interesting finding of the study was the effect of the hydrogen gas that is released as a by-product of the magnesium corrosion process. The hydrogen gas produced thin gas pockets that created an additional barrier between the soft and the hard tissues. The gas pockets formed predominantly on the upper surface of the membrane towards the soft tissue. As the gas formation occurred mostly above the membrane, bone formation was not affected. The formation of hydrogen gas during the corrosion of other magnesium implants has not been reported to have a negative effect on the long-term bone formation [30,73].

Once the magnesium metal had corroded, no more hydrogen was produced. This is evident in the 8 week and 16 week time points of the performance study. At 8 weeks post-implantation, the number and size of gas pockets have significantly reduced, and by 16 weeks, no evidence of the gas pockets can be observed.

To evaluate the corrosion mechanics of the magnesium membrane, an *in vitro* immersion corrosion test and *in vivo* corrosion kinetics study were performed. It was shown that corrosion of the membrane does not occur uniformly over its surface; instead, localized corrosion pits form that then spread and merge together over the magnesium surface. This is established in published literature and an expected outcome [59,74].

As stated in Eqs. (Eq.1), (Eq.2), (Eq.3), when magnesium corrodes in aqueous solutions, salts are formed as solid corrosion products. In-vivo, the corrosion products comprise mainly of magnesium hydroxide, magnesium carbonates and magnesium phosphates with a small number of other ions absorbed from the biological environment [75]. These form corrosion layers, the structure of which is dependent on the surrounding environment [[76], [77], [78]]. The corrosion layers were observed for the magnesium membrane in the false colored SRμCT images taken from the *in vivo* corrosion kinetics study (Fig. 7). The magnesium salts preserved the shape of the magnesium metal until they were resorbed and gradually replaced by healthy new bone, maintaining the barrier effect.

In the *in vivo* performance study, a barrier function provided by the magnesium membrane was established during the critical healing phase by the separation of the gingival tissue from the underlying defect area, after which, the membrane completely resorbed. After implantation, the membrane steadily corroded, however an occlusion of the defect site from gingival tissues was sustained for up to 8 weeks, which was adequate time for the bone to regenerate. A second phase to the functional lifespan of the magnesium membrane barrier was observed by the formation of a salty corrosion layer and local gas cavities that maintained a separation of the soft and hard tissues. After a period of 16 weeks post-implantation, the magnesium membrane had completely corroded and resorbed.

The *in vivo* performance study directly compared the clinical outcome of the magnesium membrane to that of a collagen membrane for GBR treatment in healed extraction sites. Bony defects were filled with a bone substitute material and the defect was covered with either the magnesium membrane or a collagen membrane. At every time point, inflammation at the defect site was mostly associated with the membranes due to their bioresorption processes, with variable amounts of inflammation occurring either within the gingiva away from the membrane site or surrounding the bone filler within the defect site. Therefore, it can be concluded, that after implantation, the devices are colonized by the same type and number of immune cells that induce similar levels of inflammation [79]. As the use of both membranes results in similar levels of regenerated tissue, the inflammatory response of the magnesium membrane can be judged as non-critical.

The histomorphometry results correlated with the histopathology evaluation, specifically that the bone area percentage increased with time in both treatment groups, with a greater amount in the average percentage of bone area occurring in the magnesium membrane group compared to the collagen membrane group at 1 week, 16 weeks, and 52 weeks. However, at the one week timepoint, the amount of void space was greatly increased in the magnesium membrane group when compared to the control group, which was potentially caused by the creation of gas cavities during the corrosion process. As shown by histomorphometric analysis, these gas cavities had no negative effect on tissue healing and tissue regeneration.

Therefore, it can be concluded, that both membranes have a positive healing response and their different degradation processes show no negative impact on the clinical outcome. At 52 weeks, no histological signs of either membrane were observed, and bone healing was completed to comparable levels in both groups.

## 5 Conclusion

A magnesium barrier membrane has been presented as an alternative resorbable membrane to be used in GBR surgeries. The magnesium membrane has been proven to have all of the necessary requirements for an optimal regenerative outcome from both a mechanical and biological perspective.

After implantation, the magnesium membrane separates the regenerating bone from the overlying, faster proliferating soft tissue. During the initial healing period, the membrane maintained a barrier function and space provision, whilst retaining the positioning of the bone graft material within the defect space. As the magnesium metal corroded, it formed a salty corrosion layer and local gas cavities, both of which extended the functional lifespan of the membrane barrier capabilities. During the resorption of the magnesium metal and magnesium salts, it was observed that the membrane became surrounded and then replaced by new bone. After the membrane had completely resorbed, only healthy tissue remained. The *in vivo* performance study demonstrated that the magnesium membrane has a comparable healing response and tissue regeneration to that of a resorbable collagen membrane. Overall, the magnesium membrane demonstrated all of the ideal qualities for a barrier membrane used in GBR treatment.

## Data availability statement

Data are available from the authors with the permission of Botiss Medical AG and Biotrics Bioimplants AG. The data that support the findings of this study are available from the corresponding author, FW, upon reasonable request.

## Declaration of interest

PR and ZP are employees of botiss biomaterials GmbH and FW is an employee of biotrics bioimplants AG.

## Funding sources

This research did not receive any specific grant from funding agencies in the public, commercial, or not-for-profit sectors.

## Ethical statement

We further confirm that any aspect of the work covered in this manuscript that has involved either experimental animals or human patients has been conducted with the ethical approval of all relevant bodies and that such approvals are acknowledged within the manuscript.

## CRediT authorship contribution statement

**Patrick Rider:** Writing – original draft. **Željka Perić Kačarević:** Writing – original draft. **Akiva Elad:** Methodology. **Drazen Tadic:** Methodology, Funding acquisition. **Daniel Rothamel:** Methodology, Funding acquisition. **Gerrit Sauer:** Methodology. **Fabien Bornert:** Methodology. **Peter Windisch:** Methodology. **Dávid Botond Hangyási:** Methodology. **Balint Molnar:** Methodology. **Emely Bortel:** Methodology. **Bernhard Hesse:** Methodology. **Frank Witte:** Conceptualization, Supervision, Methodology, Writing – review & editing.

## Declaration of competing interest

The authors declare the following financial interests/personal relationships which may be considered as potential competing interests:

The following authors are employees of the company biotrics bioimplants AG (Frank Witte, Marco Bartosch) and botiss biomedical AG (Zeljka Peric Kacarevic, Patrick Rider, Drazen Tadic) which companies have financed the study.

A CE mark has been successfully applied for the biodegradable magnesium barrier membrane using the published data in this manuscript.
